# Supplementary figures and images for: Homeodynamic feedback inhibition control in whole-brain simulations
Source: PLoS Comput Biol. 2024 Dec 2;20(12):e1012595. doi: 10.1371/journal.pcbi.1012595 (PMC11637364; doi:10.1371/journal.pcbi.1012595)

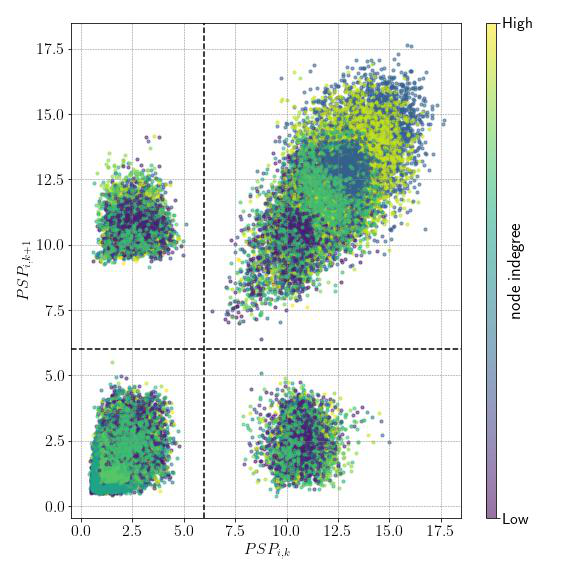

Supplement: S1 Fig — Each point (PSPi,k, PSPi,k+1) maps one PSP maximum PSPi,k of an individual node i to the next one PSPi,k+1. The node indegrees are color-coded low indegree: dark blue, high indegree: yellow. The set threshold at c = 6 mV consistently (visually) separates the FPs and LCs of the JR model. (TIF) [file pcbi.1012595.s001.tif]

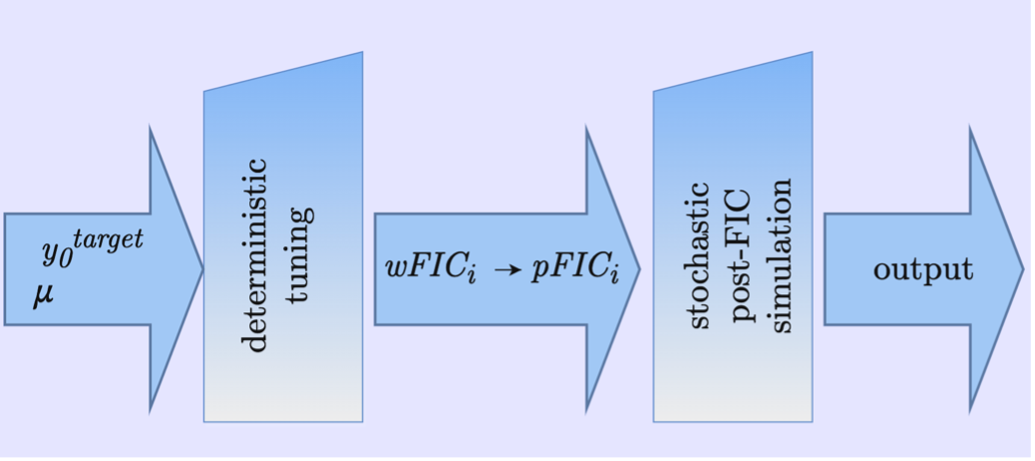

Supplement: S2 Fig — Steps: (i) μ and y0target selection, (ii) deterministic tuning resulting in wFICi vector (which needs to be checked for convergence), (iii) the last 5 seconds of the vector need to be averaged per node to obtain pFICi parameter, (iv) stochastic (optional) post-FIC simulation, (viii) simulation output. (TIF) [file pcbi.1012595.s002.tif]

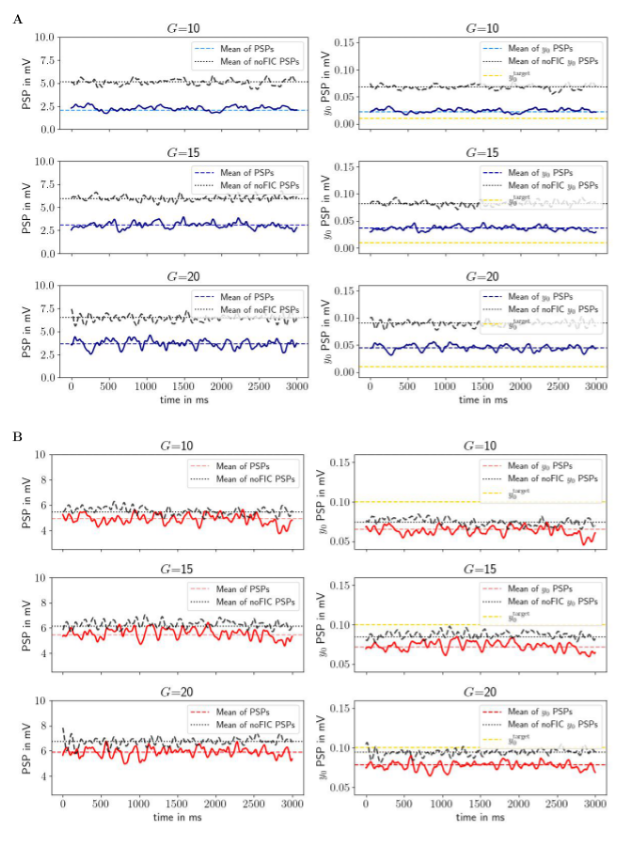

Supplement: S3 Fig — For A: y0target=0.01 and B: y0target=0.1. In both cases, the difference in the baseline of the displayed timeseries and illustrates the desired effect of tuning: In case A the dFIC limits the network-induced increase in activity despite the addition of the noise, preventing the nodes from exhibiting fast limit cycle oscillations. In case B, dFIC places the nodes at the upper boundary of bistability, allowing the system to exhibit not only fast limit cycle oscillations but also slow limit cycle oscillations–due to noise and network effects. (TIF) [file pcbi.1012595.s003.tif]

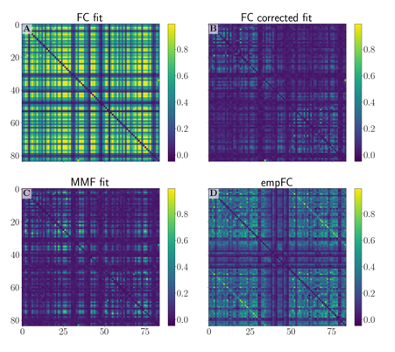

Supplement: S4 Fig — Based on pure FC correlation (top-left) and best fitting simulation after correcting for synchrony (top-right), best fitting FC matrix based on MMF measure (bottom-left) and average empirical FC (bottom-right). empFC: empirical functional connectivity; FC: functional connectivity; MMF: multi-modal factor. (TIF) [file pcbi.1012595.s004.tif]
