# Supplementary material for: Homeodynamic feedback inhibition control in whole-brain simulations
Source: PLoS Comput Biol. 2024 Dec 2;20(12):e1012595. doi: 10.1371/journal.pcbi.1012595 (PMC11637364; doi:10.1371/journal.pcbi.1012595)
Supplement: S1 Appendix — (PDF) [file pcbi.1012595.s005.pdf]

S1 Appendix

For Manusript: Homeodynamic feedback inhibition control in whole-brain simulations

Statistical analyses

We ran a statistical analysis to ensure that our simulations of BOLD signals approximate the stationary values of the MMF statistic well for each parameter set and condition (post-FIC and no-FIC simulations). We wanted to confirm that no additional simulations are needed for a robust estimation of FCs and FCDs. For each parameter combination (G, y_0^{target}) we generated a set of $N_W = 297$ MMF values computed for all overlapping time windows of $1600 * TR = 1152$ sec or 19.2 minutes, with a step size of 3 TR = 2160 ms (TR = 720 ms), drawn from the 30min original BOLD signals. Then we generated $N_s = 1000$ samples by resampling with replacement from this original set of MMF values and we computed the average MMF value across all windows for each sample to generate a bootstrapping sample distribution. Finally, we checked for the null hypothesis that the average MMF of the original set of N_W windows could have been drawn from such a bootstrapping sample distribution. **Fig A1** below depicts the respective p values for each condition and parameter combination. It can be concluded that in all cases the null hypothesis is confirmed since p generally approaches 0.5. Therefore, we consider our MMF computations robust enough.

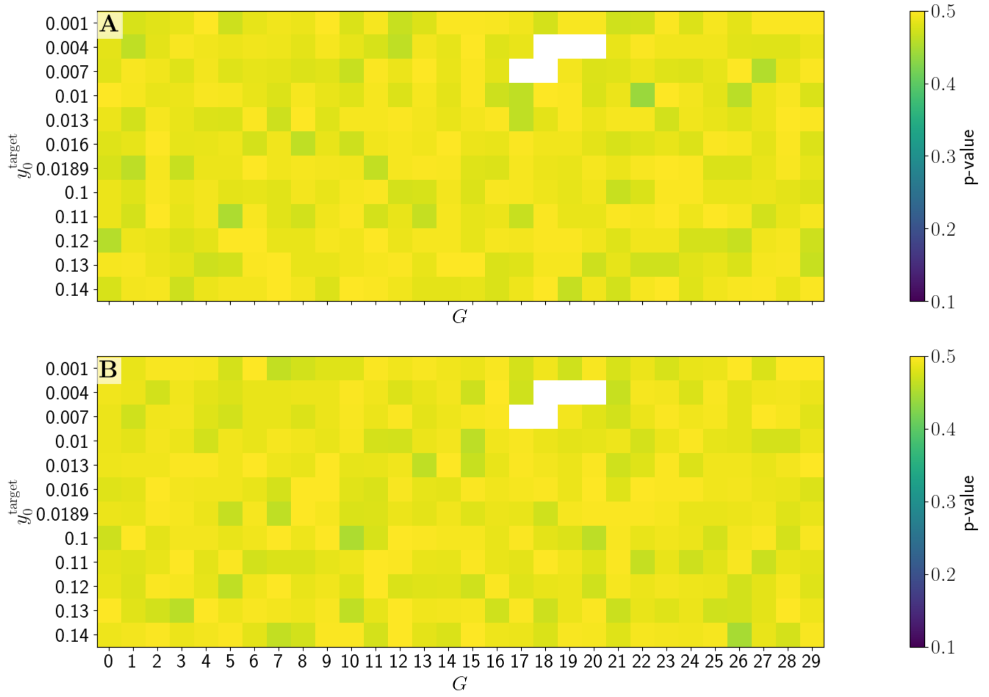

Fig A1 A-B. p-values for the null hypothesis that the average MMF values for both post-FIC (panel A) and no-FIC (panel B) simulations could have been drawn from the bootstrapping sampling distribution. The null hypothesis is confirmed for all parameter combinations, as the corresponding p-values are all > 0.1 and most of them approach 0.5, except for the parameter combinations corresponding to over-synchronized FCs, which are depicted in white color. Therefore, we conclude that the MMF computations are robust enough for both post-FIC and no-FIC simulations, already for simulated data of 19.2 minutes.

Then, we conducted another statistical analysis to estimate the significance of our differential MMF fitting results between the post-FIC and no-FIC simulations. For every parameter combination we computed the difference of mean MMF values of the above sets of N_w shorter time windows between the post-FIC and no-FIC condition. Further, we generated 1 million pairs of mixed distributions for every parameter combination, by sampling without replacement from a common set of both post-FIC and no-FIC windows and computed their respective differences of means (i.e., of the resulting pairs of mixed distributions). Finally, we checked for the null hypothesis that the difference of means across the N_w sets of MMF values could have been drawn from such a random distribution mixing the two conditions. Our results consistently demonstrate

that the null hypothesis can generally be rejected and the difference between the post-FIC and no-FIC MMF values is significant (**Fig A2**), as for almost all combinations p values < 1 million. None of the few p-values that are greater than 0.01 correspond to the regimes of interest, where the two conditions differ substantially, and particularly to any of the sub-bistable and directly super-bistable regimes identified in our work as optimal. The result is almost identical for the respective difference of the MMF values computed for the original 30 mins BOLD simulations, and therefore we do not show it here.

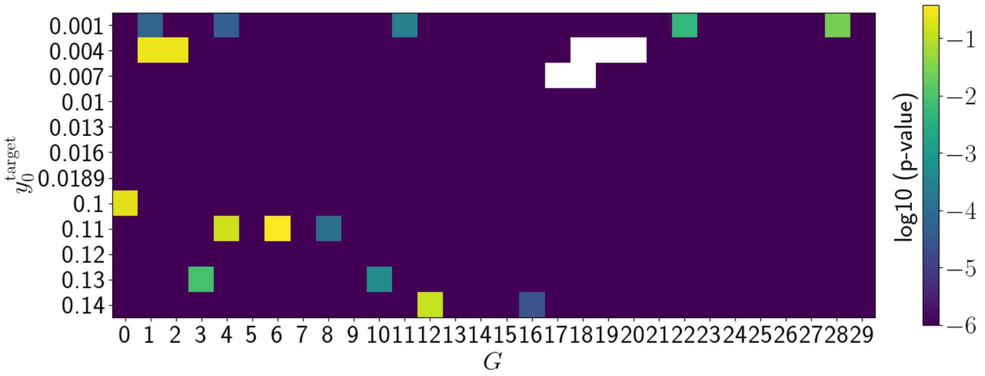

Fig A2. The heatmap of log10 (p-values) for each parameter combination based on the permutation statistical analysis. For each parameter combination we have generated 1 million random pairs of mixed distributions. Only for a few parameter combinations the respective p-value was > 0.01. Here we depict the parameter combinations corresponding to over-synchronized FCs to white color.

As a follow-up to permutation statistical analysis, we have also performed control restimulation of the main results with 15 random seeds for the entire parameter space. The initial best fits based on MMF value are within the single standard deviation of the best fits based on control simulation (**Fig A3 A-B**) for majority of y_0^{targets} for both post-FIC and no-FIC simulations. The cases when the best fitting value fell outside of this range ($y_0^{\mathrm{target}} = 0.01$ and 0.12) correspond can be explained by very low standard deviation for the control simulations and a wide range of optimal G values

yielding similar results. Additionally, we computed relative deviation for all the best fits to quantify the distance of the best fits to the mean of control simulation(**Table A1**).

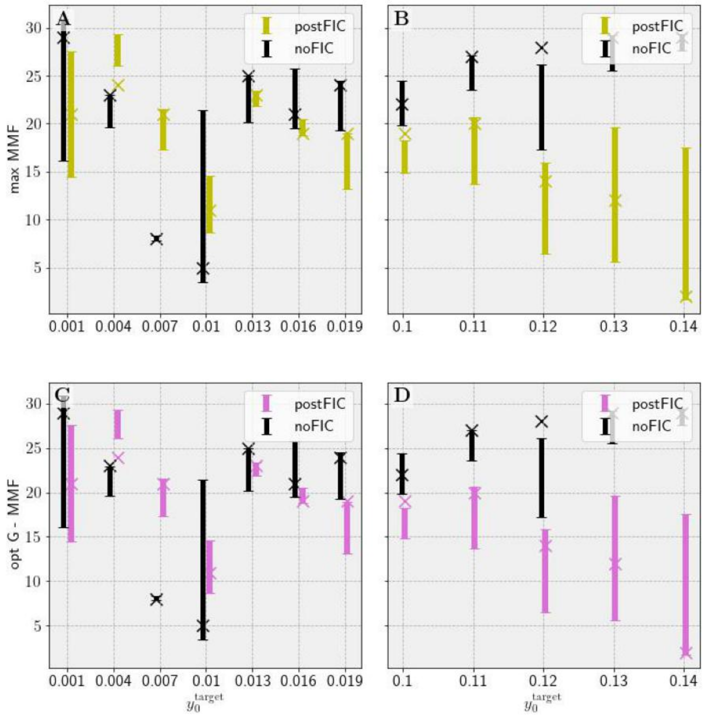

Fig A3. The results from multiple realization of the MMF fitting per y0_target (A-B) with the corresponding G values (C-D). We restimulated post-FIC and no-FIC results using random seeds (N=15). The optimal values are market with an X and error bars correspond to the standard deviation per y0_target across all simulations.

y_0^{target}	post-FIC	no-FIC
0.001	-0.0483801	0.0575306
0.004	-0.0757016	-0.0109205
0.007	-0.0187202	0.0207826
0.01	0.0280787	0.00192505
0.013	-0.0144591	-0.0202474
0.016	0.0185855	-0.00272057
0.0189	0.00056999	-0.00281066
0.1	-0.0173167	-0.00335144
0.11	-0.0254071	-0.026357
0.12	0.0947471	-0.0236518
0.13	-0.0450098	0.0381935
0.14	0.000817855	0.0317354

Table A1. The relative distances of best-fitting simulations versus best fitting control simulations (N=15).

Moreover, to test if the post-FIC simulations are significantly different from the no-FIC simulation we have computed t-test for all y_0^{targets} (**Table A2**).

y_0^{target}	T-test	p-value
0.001	-2.45182	0.02694
0.004	-9.41229	0.00000
0.007	4.01212	0.00113
0.01	60.35676	0.00000
0.013	96.74585	0.00000
0.016	35.89941	0.00000
0.0189	63.77548	0.00000
0.1	35.11730	0.00000
0.11	-42.37558	0.00000
0.12	-24.75201	0.00000
0.13	-18.84595	0.00000
0.14	-47.95023	0.00000

Table A2. T-test on the MMF values of best-fitting simulations post-FIC versus no-FIC best fitting control simulations (N=15) with the Benjamini-Hochberg correction
