# Supplementary material for: Homeodynamic feedback inhibition control in whole-brain simulations
Source: PLoS Comput Biol. 2024 Dec 2;20(12):e1012595. doi: 10.1371/journal.pcbi.1012595 (PMC11637364; doi:10.1371/journal.pcbi.1012595)
Supplement: Attachment — Submitted filename: Response2Reviewers_dFIC.pdf [file pcbi.1012595.s006.pdf]

Berlin 24.09.2024

Responses to Reviewers' Comments

Dear Editors and Reviewers,

Thank you for the opportunity to resubmit a revised version of our manuscript for publication in PLoS Computational Biology. We sincerely appreciate the time and effort that both the editors and reviewers have invested in reading our manuscript and providing valuable feedback.

We have carefully considered the reviewers' suggestions and have updated our manuscript accordingly. We believe that these revisions have significantly improved the quality of our work. Moreover, additional effort was made to improve the overall readability and conciseness of the text. To facilitate the review process, we have provided: (i) a revised version of the manuscript and (ii) a manuscript version with tracked-changes, and (iii) this point-by-point response to each comment below (reviewers' comments in blue font with our answers in black font). Each significant change in the main text resulting from reviewers' comments is explicitly mentioned in our responses in this document, with the exception of the significantly shortened introduction which has been rewritten to address a number of reviewers' comments and can be found in the tracked-changes version of the manuscript.

We are grateful for the opportunity to improve our work based on the suggestions provided by the reviewers. We look forward to your further consideration of our revised manuscript.

Best regards,

Jan Stasinski (on behalf of all co-authors)

Reviewer #1:

We appreciate reviewer's positive comments and suggestions.

R1.C1

The authors show that this method can control the target activity level to obtain biologically plausible neural activity, including central features of BOLD signals. Interestingly, the method is also shown to maintain to high variability of model dynamics corresponding to critical/bifurcation points hypothesized to be important for brain function. Although presented mainly as a technical solution to stabilize large-scale brain simulations, the work also adds support to the hypothesis that neural homeostasis through inhibitory plasticity may be functionally important - I wonder if this idea could be explored in a data-driven way with the methods presented here.

Authors' Response:

We agree with the reviewer's comment. We addressed it by mentioning recent studies in the Discussion that stress the importance of the excitation–inhibition balance in the brain and suggest inhibitory synaptic plasticity as a possible mechanism for the brain to maintain such

a balance in the clinical context (Coronel-Oliveros et al 2024, Moguilner et al., 2024) Starting at the line 834: *"The mechanisms related to inhibitory control, similar to dFIC, have potential relevance to modeling E-I imbalance in the clinical context. Parameters responsible for the balance between excitation and inhibition have been linked to abnormalities in Alzheimer's Disease, such as synaptic degeneration, hyperexcitation, white matter atrophy, as well as amyloid-beta and tau levels. In previous studies, inhibitory parameters of BNM were fit to empirical functional data of patients (9,84). Recently, the JR model has been used to derive biomarkers related to inhibitory control, emphasizing the potential of model-based approaches in the early diagnosis of dementia and Alzheimer's disease (57,85). Similarly, the role of inhibitory control in the progression of dementia has been studied using the Wilson-Cowan model (86) and the Deco model from 2018 (87,88). As we have shown, dFIC improves the overall model fitting, facilitating the study of how different dynamical regimes and transitions between them may be linked to various pathological conditions. Additionally, the resulting inhibitory plasticity parameters, can be interpreted as measures of dynamical differences in clinical setting."*.

Since inhibitory feedback is thought to be the main mechanism of controlling for local overexcitation, there exists a solid basis for investigating this subject further and the here presented dynamic Feedback Inhibition Control (dFIC) can be used for such a project (i.e Abeysuriya et al. 2018, Moguilner et al., 2024).
The fact that FIC improves the fit with FC and firing rate distributions already points towards functional importance. However, in-depth exploration of the mechanism's functional importance in a data-driven way is an idea we would like to pursue further in future work, as it remains outside the scope of this paper, which is methodological in nature. Indeed, ongoing work in our laboratory aims at fitting brain parameters heterogeneously, at the level of each brain region (i.e., one parameter per node in the TVB brain network model), against FC and FCD empirical data, using Bayesian methods. An alternative promising hypothesis would be that such heterogeneity across the brain network could result from local inhibitory plasticity, functioning in a way similar to dFIC, to constrain the overall activity of the brain within limits. Our ongoing work aims at comparing these two model hypotheses for their efficiency to fit empirical data, taking into consideration model complexity as well.
Our dFIC equations in the present paper are designed, though, to facilitate their analytical treatment and ensure convergence to the activity target. A more in-depth study of the role of inhibitory plasticity might require their further specification with a more biologically realistic mathematical model of inhibitory plasticity.
We have added a brief reference in the Discussion to studying inhibitory plasticity as a homeodynamic mechanism for fitting FC and FCD data in future work in line 893: *"Further research can also investigate dFIC as a homeodynamic mechanism, possibly relying on inhibitory plasticity, which maintains the state of the brain within limits and responds to external perturbations induced by e.g., cognitive stimuli. This kind of slow timescale modulation of brain dynamics could be of great interest in understanding data, e.g., on FCD dynamics of BOLD signals."*

R1.C2

I think though that equations 1-4 miss the dynamics (it looks like eqn. 4c should be the derivative of wFIC).

Authors' Response:

The notation chosen for equations uses a dot above the differentiated variable which is present in Equation 4 (c). We hope that this clarifies the reviewer's concern.

R1.C3
Second, it would be prudent to document the published code to enable re-use. As it is, the repository only has some python files without any documentation or instructions.

Authors' Response:

A README.md file and improved documentation of the code has been added to the referenced GitHub repository:
https://github.com/BrainModes/dFIC_code

Reviewer #2:

I would like to recommend this article for publishing only after significant efforts would be made for improving the quality of manuscript.

Authors' Response:

We thank the reviewer for the comments, suggestions and pointing us to the mistakes. We did our best to address them.

R2.C1
Overall readability should be significantly improved.
We carefully revised the manuscript focusing on readability, and we feel this has significantly improved the manuscript.

R2.C1.1
There is substantial number of honest mistakes across the whole paper. For instance, reference 18, 19, 59, 72, 84, 92 were obviously broken. Equation 6 was not indexed. At line number 485, referring figure number went wrong. I could find a dozen more even after initial superficial reading of the manuscript, and it is hard to list all that I found while repetitive readings. It could be simply minor points to improve but many mistakes arouse a doubt that there might be overlooked critical ones. I would like to recommend that the authors thoroughly check especially, figure, equation, reference number in the text.

Authors' Response:

We have corrected the references and the figure as well as equation numbering and thoroughly proof-read the entire manuscript. We hope that the overall quality has significantly improved.

R2.C1.2
Several acronyms were not introduced properly. Ex) JR at line number 152, FC at 164.

Authors' Response:

This issue has been noted and addressed by changes to respective lines.

R2.C1.3
All the coefficients and variable names should be at least once briefly explained. Ex) T in eq 5a, R_FC in eq 6.

Authors' Response:

This issue has been addressed by adding a clarification in lines 286 and 364.

R2.C1.4

Table 1 was never referred in the text. In addition, though JR model and its coefficient set are well-known, the supporting prior works or reasoning to determine the values should be offered in the text.

Authors' Response:

Indeed, the reference to Table 1, which in the submitted version was only present in the captions of Fig 2 and Fig 4. We have added a reference to the main text in the line 179 including the introduction of model parameters and their values: *"The parameter values used for simulations are listed in Table 1 and based on physiological considerations in the original Jansen and Rit publication (52). The amplitudes of the EPSP and IPSP are determined by $A = 3.25$ mV and $B = 22$ mV for the excitatory and inhibitory cells, respectively. The corresponding durations of EPSP and IPSP are inversely proportional to $a = 0.1$ ms^{-1} and $b = 0.05$ ms^{-1}. As in the original work (52), the firing threshold is given by v_0= 6 mV. The remaining parameters of the sigmoidal transfer function in Eq (2) were set at v_max=0.0025 s^(-1), e_0= 2.5 s^{-1} and $r = 0.56$ mV^{-1}. The constant J=135 corresponds to the number of the synaptic contacts, which is scaled for individual connections with c_1,c_2,c_3 and c_4."*

R2.C1.5

'Introduction', especially line 74-126 or line 127-188 can be substantially shrunk by removing redundant messages and rearrangement to 'discussion'. The reason why I brought up this issue is that because of this long introduction compared to the very specific and neat motivation and summary of the work, the main novelty gets blurred, resulting in unnecessary complexity of the flow.

Authors' Response:

We reworked the main part of the Introduction starting from line 72 shortening the text, removing the redundancies and moving relevant snippets of text to other sections. Two other comments by the reviewer 3 suggested shortening and improving the introduction: - R3.C1.1 and - R3.C1.2. In this revision we incorporated the suggested changes in one step as the message in all three comments was consistent. We are providing now the reviewers and editors with a detailed view of all the changes made to the Introduction via the manuscript with tracked-changes.

Before the revisions the Introduction had 2045 words, whereas the current version has 1325 words.

R2.C2

Since the authors emphasized that the introduction of dFIC into JR model improves the pipeline to construct a better BMN regenerating 'biologically plausible' brain signal, the novelty of this work was spotlighted mainly in the perspective on 'observation-simulation matching.' Although JR model and the other types of mean-field models don't target the biological plausibility (or similarity) of local network architecture, dFIC seems capable of somewhat compensating such simplification. I would like to recommend for the authors to state this point briefly. Although somehow, in Discussion, the section stating E-I balance is weakly related to this point, it seems possible that a bolder interpretation, such as dFIC can be the biological mechanism of local E-I balance, can be suggested.

Authors' Response:

We appreciate the reviewer bringing up this subject. We agree that both the ability of the inhibitory control to reflect the biological mechanisms of E-I balance, and the clinical relevance of such a relation mentioned in the next comment (R2.C3) deserve to be addressed in the manuscript. In response to it, the comment below and another comment by the reviewer 3 (R3.C1.8), we have added a paragraph in the line 846:
"To sum up, dFIC is designed to maintain the activity of all nodes at a certain level, by adjusting local inhibition and, in that manner, maintain E-I balance at both local and - indirectly - at the global scale regardless of the chosen target. Our exploration of different activity targets suggests that tuning the system to the proximity of bistability results in increased switching between regimes and better fits to the empirical data. dFIC provides improvement in characteristics observed in the empirical signals."
We strived to keep this section concise to serve as a short summary of efforts in that direction and as an outlook for future work.

We have refrained from specifically equating the dFIC with biological mechanism of local E-I balance, but decided to add the following clarification at like 798:
"It is worth noting that several mechanisms collectively contribute to the brain's ability to self-regulate the E-I balance through synaptic plasticity (80,81). Recent studies suggest the roles of short-term (82) and long-term synaptic plasticity (83), and structural plasticity (84) in maintaining this balance. Additionally, there are cellular mechanisms distinct from plasticity, but with capability of self-regulation. As an example, spike-frequency adaption, i.e., the property of certain types neurons to reduce their firing frequency upon sustained input, could effectively act as a negative feedback similar to feedback inhibition (85). Overall, mechanisms like dFIC are implementations of generalised inhibitory synaptic plasticity mechanisms in the BNMs. However, their effectiveness in maintaining the E-I balance in simulated networks does not allow for determining the specific biological underpinnings of such mechanisms."

R2.C3

As the extension of 2, it would be much better to be discussed, the determination of wFIC and pFIC in the pathological conditions (one of the main purposes of TVB) and the implications of resultant wFIC and pFIC.

Authors' Response:

Indeed, there have been recent efforts to relate the inhibitory control parameters to pathological conditions. We have included two such examples in our short addition to the Discussion, mainly the work of Ranasinghe et al., 2022, Martinez-Cañada et al., 2023, Moguilner et al., 2024. We hope that this addition, albeit brief, addresses the reviewer's suggestion (line 833): *"The mechanisms related to inhibitory control, similar to dFIC, have potential relevance to modeling E-I balance also in the clinical context. Parameters responsible for the balance between excitation and inhibition have been linked to abnormalities in Alzheimer's Disease, such as synaptic degeneration, hyperexcitation, white matter atrophy, as well as amyloid-beta and tau levels. In previous studies, inhibitory parameters of BNM were fit to empirical functional data of patients (9,84). Recently, the JR model has been used to derive biomarkers related to inhibitory control, emphasizing the potential of model-based approaches in the early diagnosis of dementia and Alzheimer's disease (57,85). Similarly, the role of inhibitory control in the progression of dementia has been studied using the Wilson-Cowan model (86) and the Deco's model from 2018 (87,88). As we have shown, dFIC improves the overall model fitting, facilitating the study of how different dynamical regimes and transitions between them may be linked to various pathological conditions. Additionally, the*

resulting inhibitory plasticity parameters, can be interpreted as measures of dynamical differences in clinical setting."

R2.C4

Poincare map

R2.C4.1

In Figure 2, I did not understand how Poincare map without varying lag time or transition map (between dots) could specify the dynamics of each regime such as slow limit cycle or fast limit cycle.

Authors' Response:

We agree, the previous description of our approach to Poincaré analysis was partially incomplete. Thank you for pointing out this issue. We have used Poincaré section based on local maxima of the signals of each node as a basis for creation of maps of lower dimensionality. We admit that for the exhaustive Poincaré analysis it would be appropriate to vary the time lag. For our purpose of understanding basic dynamics in terms of the exhibited regimes using a single time lag of 100ms was deemed sufficient. The time-series simulated with the Jansen-Rit model are complex but, naturally, not as complex as empirically obtained brain signals. We have also extended the method section dedicated to Poincare analysis methodology as suggested by the reviewer (line 347): *"..We implemented a method for a Poincaré mapping involving the analysis of a PSP time-series (68–70). Poincaré maps are derived from the Poincaré sections, representing a projection of a dynamical system's behavior onto a lower-dimensional space. Here we take the simulated PSP timeseries $PSP_i(t)$ of region i and determine the k-th local maximum $PSP_{i,k}$ numerically by comparing each value at a given time point with neighboring points in a 100 ms time window. The first recurrence map given by $PSP_{i,k} \rightarrow PSP_{i,k+1}$ yields a point cloud in which each point corresponds to state changes of individual nodes. The method essentially maps the continuous dynamical system into a discrete one, acting like snapshot of changes in the maxima of the signal. Nodes which stay nearby an attractor, be it FP or LC, exhibit fluctuations around that attractor due to noise. Consequently, the points $(PSP_{i,k}, PSP_{i,k+1})$ spread around the attractor – for FP around the PSP value of the FP and for LCs around all PSP value of local maxima within one period."*

R2.C4.2

In the caption of fig2, 'each color marks a single node' and 'colors on the map mark the indegree of the nodes' are very confusing comments.

Authors' Response:

Indeed, the caption in the previous version was unfortunately confusing. We have also decided to place original Figure 2 in the Supporting Information, in the name of conciseness and overall clarity of the manuscript. The caption has been adjusted for clarity:

Caption of S1 Fig states now: ***"S1 Fig. Example Poincaré map of the PSP from a single simulation****. Each point ($PSP_{i,k}, PSP_{i,k+1}$) maps one PSP maximum $PSP_{i,k}$ of an individual node i to the next one $PSP_{i,k+1}$. The node indegrees are color-coded (low indegree: dark blue, high indegree: yellow). The set threshold at $c = 6\ mV$ consistently (visually) separates the FPs and LCs of the JR model."*

Additionally, for consistency, we have added: This revised caption starting from line 706: *" Fig 15. Poincaré maps for the best no-FIC (A1) and post-FIC (B1) MMF fit. Each point* $(PSP_{i,k}, PSP_{i,k+1})$ *maps one PSP maximum* $PSP_{i,k}$ *of an individual node* i *to the next one* $PSP_{i,k+1}$. *The node indegrees are color-coded (low indegree: dark blue, high indegree: yellow)..."* to the Results, to the caption Fig 15.

R2.C4.3

As an extension, it would be better to kindly offer the full details of the Poincare map analysis in Method section.

Authors' Response:

Following up on previous comments pertaining to the Poincare maps we have extended the respective methods section.
Please see response to R2.C4.1.

Additionally, a more exhaustive description of the reasoning behind the Poincare maps analysis has been added to the Results at the line 667:
"First, we have identified a threshold of activity ($c = 6\ mV$) *which can be applied to the resulting maps to identify the regime of the JR model being exhibited by any node at any given time. More in detail, the lower activity FP of JR in presence of noise, yields points* $(PSP_{i,k}, PSP_{i,k+1})$ *in* $[0\ mV, 6\ mV] \times [0\ mV, 6\ mV]$, *the fast LC in* $[6\ mV, 18\ mV] \times [6\ mV, 18\ mV]$, *whereas one period of the slow LC will have one point in* $[0\ mV, 6\ mV] \times [6\ mV, 18\ mV]$ *and one in* $[6\ mV, 0\ mV] \times [0\ mV, 6\ mV]$, *due to the formation of two maxima within one period. If a node traverses multiple regimes however, the point cloud of a single node is going to fulfill multiple of those conditions, allowing to identify which regimes were visited and therefore which dynamics exhibited. By analysis of the pattern formed by individual nodes over time and the pattern of all nodes together, it is possible to extend the understanding of the overall behavior of the model. Further the nodes were classified according to the number of dynamical regimes each node 'visits' for more than 7,5% of the simulation time* T_{post}*."*

R2.C4.4

In Figure 15 in the subplots, traversing number 3 seems not changing in any case (~25). I would like to recommend that the authors offer the interpretation on it.

Authors' Response:

Indeed, Figure 15 requires additional information and interpretation. We thank the reviewer for pointing this out. In the revision we improved the plot by adding the percentage count on top of the bars in the subplot to demonstrate more precisely the effect of FIC. This way the change in exhibited regimes per node is more apparent.

Importantly, we expected FIC simulations to improve the overall richness of behavior exhibited by the model not necessarily limited to the increase of nodes exhibiting all three regimes. What we considered an improvement was the decrease in number of nodes in a single regime or increase in the number of nodes in two or three regimes. Depending on factors including but not limited to: y_0^{target}, global coupling noise, network effects and delays the availability of different regimes can vary and FIC is not sufficient to precisely control all individual nodes. We hope that the edited Figure 15 underlines this argument better now.

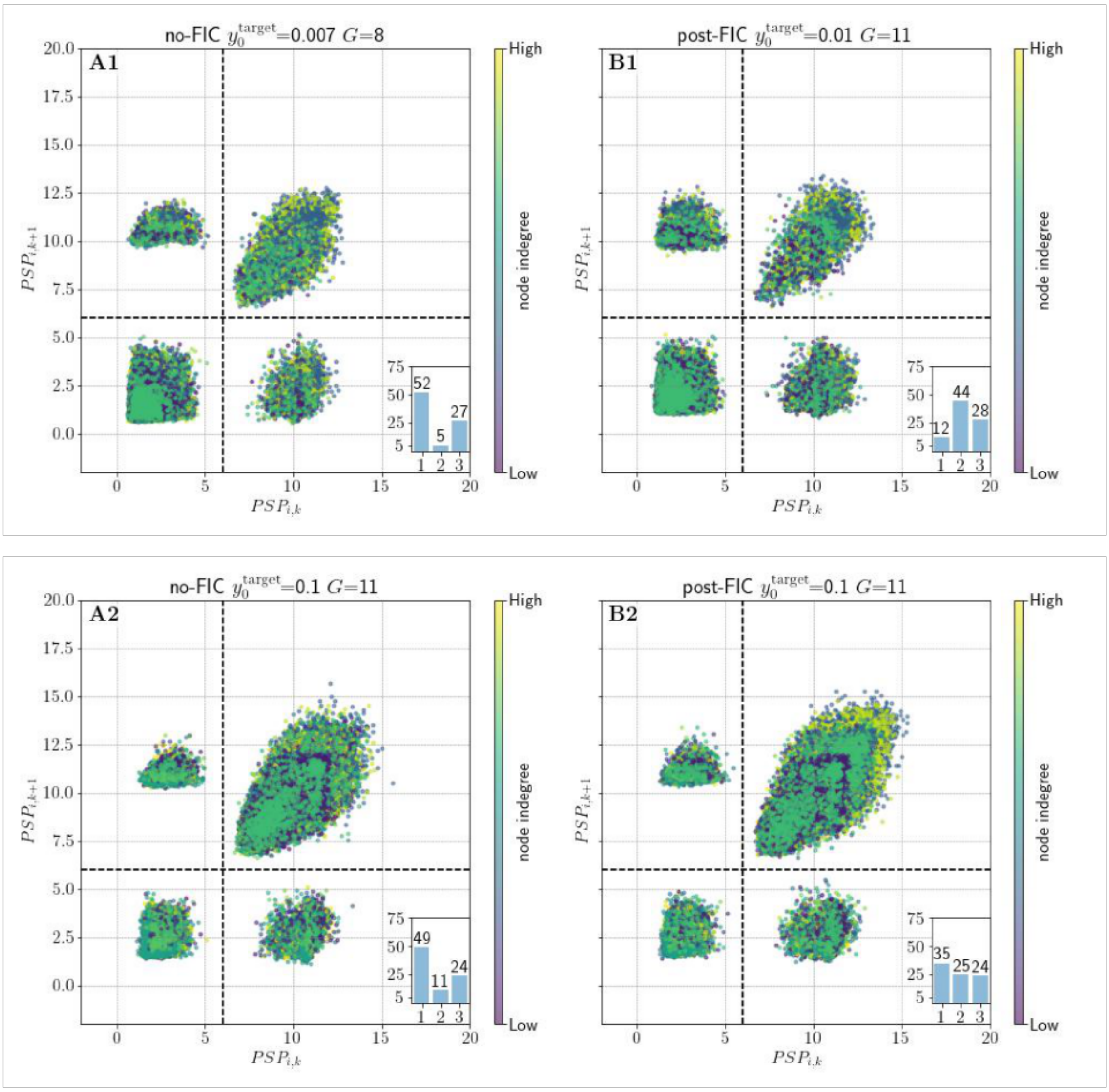

Reviewer #3:

I would recommend this manuscript if the authors can address the following major and minor suggestions.

We thank the reviewer for the time and effort put in the comments and suggestions below. We believe that they contributed to the overall improvement of the manuscript.

Majors

R3.C1.1

The article would benefit from a reduction of content, especially in the Introduction section, which is quite long. For example, the paragraph that starts in line 97 "from a dynamical systems perspective..." is redundant compared to the one before it. The two paragraphs of lines 114 and 127 are both about E/I balance, hyperexcitability, and FIC, and can be reduced and combined into one.

Authors' Response:

Following these recommendations and similar ones of two additional reviewers (R2.C1.5 and R3.C1.2), we revised and shortened the Introduction. Please see our above responses to R2.C1.5 and R3.C1.2.

We also have reworked parts of Methods and Discussion to address all the comments from reviewers.

R3.C1.2
The paragraph of line 143 can be reduced or removed. Other phrases, like "This version of FIC is formulated as a set of ordinary differential equations..." (line 180) can be moved to Methods, and other ones like "The analytical treatment of the single-node tuning dynamics reveals the conditions..." (line 185) may be more properly placed in the Results.

Authors' Response:

Please see our response above (R3.C1.1).

R3.C1.3
What is the motivation of the authors for using three differential equations for including the FIC mechanisms, instead of just one (like in Abeysuriya et al., Plos Comp Biol, 2018)? For me, it wasn't clear when reading the text. What is the advantage of including the additional two state variables in the model? Perhaps the authors can address this point in the Discussion, and/or compare the model's outputs or performance with a version of the model that includes only the equation 4c.

Authors' Response:

This important question, was indeed, not fully elaborated in the paper: The purpose of the state variable described by the equation 4a is to obtain a time-averaged slow tracking of the ongoing firing rate as fitting target, which ensures that $wFIC_i$ variable doesn't fluctuate or oscillate. In cases of a fixed-point target, such averaging filters out noisy fluctuations, whereas in cases of limit cycles, averaging sets the center of the limit cycle as the target of the learning equation.
In Abeysuriya et al. the baseline excitatory activity is set at the fixed point ($P = 0.31$) right before the Hopf bifurcation, and the relatively low ISP target ($p = 0.15$) corresponds to the oscillatory regime, similarly to our work. This effectively means that the ISP state variable has to oscillate around the target. The authors implement time averaging by selecting a large enough time constant (τ_{isp}) for the learning dynamics. Moreover, they employ an additional time averaging equation for the criterion of convergence to the target of learning (target excitatory activity ρ in equation 4 of the main text), which is though provided only in the Supporting Information (equation A2). There, they also discuss the effect of this averaging on the learning target, in order to justify the specific weighting of the excitatory activity with the inhibitory one, which they employ in their averaging equation.
In our work we decided to follow Vogels et al. 2011 and implement the slow averaging equations explicitly, despite the fact that the inclusion of two more equations increases the complexity of the system. We did so firstly so that we provide the analytical treatment in the form presented in the manuscript, based on the explicit separation of fast and slow timescales; secondly, so that the slow averaging is disassociated from the exact form of the learning equation (see below).
Therefore, one could indeed substitute the slow averaging with a smaller learning rate as Abeysuriya et al. Depending on the learning equation, e.g., its (non)linearity, this solution could be sufficient. In other cases, though, our simulations showed that such an option could lead to either of the following undesirable results: (a) a larger learning rate (η in our case)

generally increases the simulation time for convergence of dFIC, and (b) the resulting effective excitatory activity, once dFIC is turned off, might be different than the intended target to a degree again depending on the learning rate, as we observed in the case of our nonlinear learning equation. Further systematic exploration would be needed to investigate the interaction between any concrete combination of neural mass and learning equations to clarify this issue. By including the slow averaging detection equations, we try to disentangle the two systems. We have added a sentence of clarification in the line 260:

"We chose to include such equations to facilitate our analytics by making fast and slow time scale separation of the dFIC system more explicit, as well as in order to disassociate the slow averaging dynamics from the particular form of the learning dynamics, and given that some slow time averaging is in all cases necessary to filter out fast noisy fluctuations (in the case of point attractors' targets) or oscillations (in the case of limit cycle y_0^{targets}). The Eq 4a provides a time-averaged slow tracking of the ongoing firing rate as fitting target, which ensures that $wFIC_i$ variable doesn't fluctuate or oscillate."

R3.C1.4

The authors discarded simulated FCs with a mean > 0.25. This can be avoided using metrics that "balance" connectivity pattern similarity and differences in global correlations, like the structural similarity index (like in Ipiña et al., 2020, Neuroimage).

Authors' Response:

We agree with the reviewer on the need to clarify this point. As the main focus of the paper is the inhibitory mechanism itself and its consequences, we decided to use the average of the empirical FC threshold for simulated FCs as a constrain, not as the optimization target. Following the reviewer's suggestion, we computed the SSIM fits and obtained almost identical results; the best fitting simulation based on Pearson correlation of FCs was: $y_0^{\text{target}} = 0.007$ and $G = 18$; based on Pearson correlation of FCs with threshold: $y_0^{\text{target}} = 0.007$ and $G = 21$, and for the SSIM: $y_0^{\text{target}} = 0.007$ and $G = 19$ (**Fig. RV1**). The overall parameter landscape did not change significantly when different methods were applied. Nothing stands against using a different fitting method. For us using a threshold was dictated by simplicity and aimed at excluding the biologically unrealistic FC (**S4 Fig panel A** can serve as an example) of too high synchrony, by using the average of empirical FC as a reference.

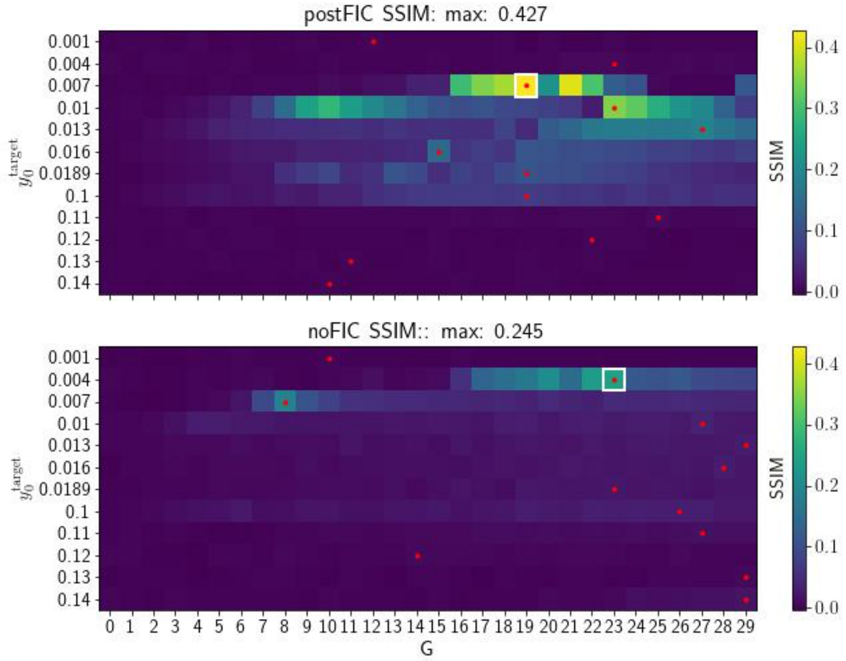

Fig RV1. The heatmaps showing SSIM(FC) fits.

R3.C1.5

Why the authors didn't use the SSIM or other composed metrics (similar to Pfeffer et al. 2021, Science Advances)?

Authors' Response:

Indeed, other metrics, including SSIM can be used for fitting in this setting. As noted, in response to comment R3.C1.4, we followed up on the reviewer's suggestion and computed both the SSIM and the compound measure explicitly using the distance between average empirical FC and each of the simulated FCs in addition to Pearson correlation between those matrices Fig RV2) as in suggested work by Pfeffer et al. 2021. What we found in both cases is that the change in the metric does not yield significantly changed optima of fit (y_0^{target} remains the same but the optimum changes from $G = 18$ to $G = 19$). Both of those measures, however, fail to reject the FC matrices with clusters of regions with high synchrony (Fig RV3). Thresholding at the level of empirical FC synchrony and the linear combination of Pearson correlation and KS-distance between FCD distributions has been proven to prevent the inclusion of over-synchronized FCs. Although we concede that the usage of more advanced continuous metrics can be beneficial in the future, for the purposes of this work we decided to use the simple approach with thresholding and linear combination of weighted measures.

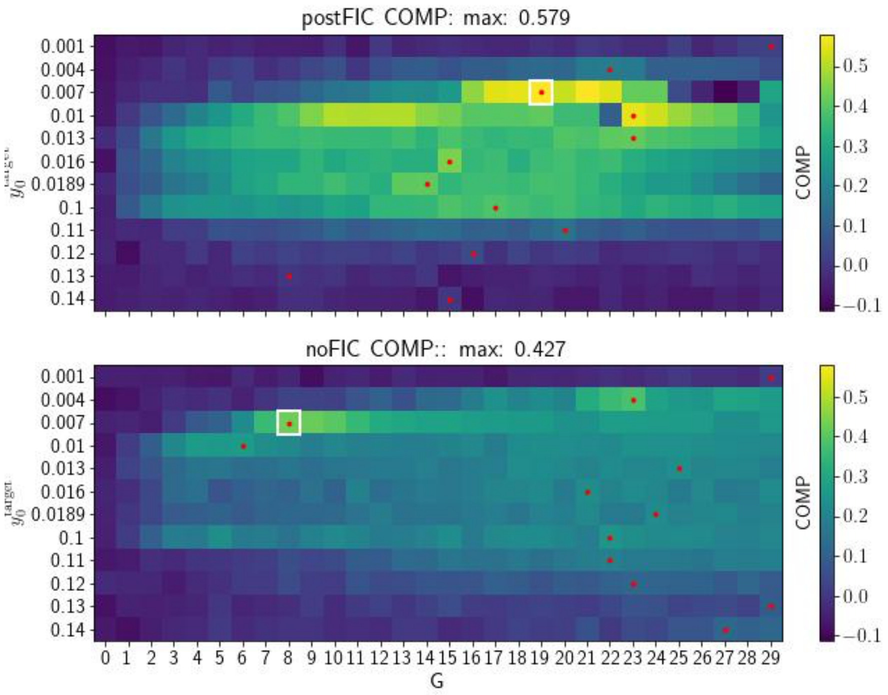

Fig RV2. The heatmaps showing the compound measure using the distance between average empirical FC and each of the simulated FCs subtracted from respective Pearson correlation between those matrices.

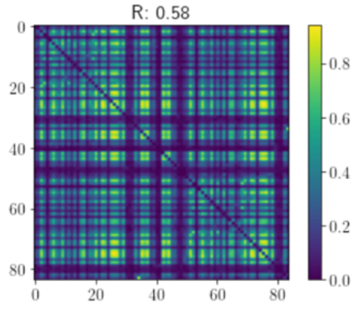

Fig RV3. The simulated FC matrix for $y_0^{\text{target}} = 0.007$ and $G = 19$

R3.C1.6

The authors showed that the model can fit fMRI BOLD FC and FCD. However, it would be good to show that the model is also able to fit some properties of empirical EEG (e.g., EEG FC, power spectrum). The authors can try to fit both fMRI and EEG dynamics using the model and the data from the HCP. If not, it would be nice at least to see what the EEG power spectrum looks like when using the optimal parameters of the fMRI fitting. I also recommend addressing this limitation of the work in the Discussion if not addressed.

Authors' Response:

The reviewer raises a valid point about the potential improvement coming from including fitting to empirical signals with high temporal resolution. However, extending this work to another modality goes beyond the scope of this paper. For the purpose of assessing the effects of dFIC on the postsynaptic potentials we decided to limit our methodology to the analysis of postsynaptic potentials in different conditions presented in Figs 8,14,15. Besides, ongoing work in our laboratory aims at using Bayesian methods of model inversion to fit TVB models against multimodal data (EEG/MEG and BOLD).

Following the reviewer's suggestion this paragraph was added to the limitations' section of the Discussion at the line 884:

'Our work utilized fitting different properties of the simulated BOLD signals to assess their 'biological plausibility', including FC, FCD and synchrony. However, it did not extend to the high-temporal resolution signals such as MEG and EEG. Even though, we investigated the basic properties of the generated post-synaptic potential time-series (with and without dFIC), in the frequency domain and via Poincare Maps, we did not extend our fitting procedures to include those other signal modalities (Figs. 8,14,15). Such multi-modal fitting can, in the future help determine the relationship between different modalities and further improve both the fitting methods and the homeodynamic fitting procedures.'

We have also updated Figure 8 to exclude the no-FIC simulations matching the best fitting post-FIC simulations to better highlight the difference in power spectra between best fitting post-FIC and no-FIC simulations for both Pearson correlation between FC matrices and the combined MMF measure. It is worth noting that both measures pointed to the sub-critical regime as optimal based on the properties of the BOLD signals. So, they do not necessarily guarantee a close match with well-known resting state EEG/MEG power spectra.

R3.C1.7

In the Introduction section, Line 152, the authors said that "... so far, an FIC mechanism is missing for the Jansen & Rit model". Indeed, a mechanism for preserving the E/I balance in the Jansen & Rit model was proposed by Coronel-Oliveros et al. (2024, Alzheimer's & Dementia). I think that the authors should mention this paper as an antecedent of FIC mechanisms in the Jansen & Rit model. The mechanism proposed in Coronel-Oliveros et al. is very similar (but of course not equal) to the implementation proposed by Stasinski et al. here, without considering the additional activity detection equations (4a and 4b). Perhaps the authors can discuss the differences between these two versions of the Jansen & Rit model.

Authors' Response:

The reviewer is right pointing out the missing reference to Coronel-Oliveros et al. (2024, Alzheimer's & Dementia). It is indeed true that the mechanism proposed there is similar, i.e., a slightly simplified version of the dFIC presented in our work. The role of the activity detection equations has been elaborated on in response to comment R1.C1.3 referring to the number of equations used for the FIC mechanism. Perhaps it is worth noting that one goal of our work is to study the mechanism itself to better understand the effects and limitations of this method via analytical methods and using a variety of tuning targets. For this purpose, we chose to include the activity detection equations to provide a broader overview of the method's capabilities. We have included a sentence in the edited introduction in Line 125:

"So far, the only known implementation of the FIC in the JR model comes from the recent work Coronel-Oliveros et al., 2024... ".

R3.C1.8

It would be a nice addition to the Discussion to talk about possible clinical applications, in a very short paragraph, of the JR + FIC model, for example, proposing how the FIC can be used to study E/I disbalances in dementia (see for example Ranasinghe et al., 2022, eLife; Martinez-Cañada et al., 2023, Alzheimer's & Dementia: Diagnosis, Assessment and Disease Monitoring; Coronel-Oliveros et al., 2024; Alzheimer's and Dementia; Moguilner et al., 2024; Alzheimer's Research & Therapy). In this other work, the authors used the JR model for proposing biomarkers from the model, and not from the data: Amato et al., 2024, Alzheimer's & Dementia: Diagnosis, Assessment and Disease Monitoring).

Authors' Response:

Following the reviewer's suggestion we have added a short section dedicated the clinical outlook related to the inhibitory control in the line 833:

"The mechanisms related to inhibitory control, similar to dFIC, have potential relevance to modeling E-I balance also in the clinical context. Parameters responsible for the balance between excitation and inhibition have been linked to abnormalities in Alzheimer's Disease, such as synaptic degeneration, hyperexcitation, white matter atrophy, as well as amyloid-beta and tau levels. In previous studies, inhibitory parameters of BNM were fit to empirical functional data of patients (9,84). Recently, the JR model has been used to derive biomarkers related to inhibitory control, emphasizing the potential of model-based approaches in the early diagnosis of dementia and Alzheimer's disease (57,85). Similarly, the role of inhibitory control in the progression of dementia has been studied using the Wilson-Cowan model (86) and the Deco's model from 2018 (87,88). As we have shown, dFIC improves the overall model fitting, facilitating the study of how different dynamical regimes and transitions between them may be linked to various pathological conditions. Additionally, the resulting inhibitory plasticity parameters, can be interpreted as measures of dynamical differences in clinical setting. We thank the reviewer for his helpful comment and the relevant list of references to consider.

Minors

R3.C2.1

Line 26 in Abstract: "Using a variety of metrics". What metrics?

Authors' Response:

Following the reviewer's comment we have rephrased this part of the abstract and decided not to mention specific metrics at that early stage. Now abstract from line 26 states: *"We demonstrate that the derived dynamic Feedback Inhibitory Control (dFIC) can be used to enable increased variability of model dynamics. We derive the conditions under which the simulated brain activity converges to a predefined target level analytically and via simulations. We highlight the benefits of dFIC in the context of fitting the TVB model to static and dynamic measures of fMRI empirical data, accounting for global synchronization across the whole brain. The proposed novel method helps computational neuroscientists, especially TVB users, to easily "tune" brain models to desired dynamical regimes depending on the specific requirements of each study. The presented method is a steppingstone towards increased biological realism in brain network models and a valuable tool to better understand their underlying behavior."*

R3.C2.2

Line 30-31 in Abstract: the two lines might be reduced to "... of fitting simulated to fMRI static and dynamic functional connectivity data".

Authors' Response:

We have removed *"...with a comparison of two separate fitness functions: static functional connectivity correlation and a multimodal function..."* from the abstract as suggested.
Please also see response to R3.C2.1.

R3.C2.3

The Figure 1 description (and the figure itself) should be moved to the Results section.

Authors' Response:

We appreciate the reviewer's suggestion and see the reason in placing such description in the results. Even though the Figure 1 is a previously established result and not our original result, it is not uncommon to place such information at the beginning of the results section. We

decided to leave the description of the regimes and introduction of the bifurcation structure of the Jansen-Rit model in the Methods section for two reasons: First, in our opinion Figure 1 and the section entitled “Regimes of the JR model” serve as methodological introduction to a rather complex behavior of the model and provide reader with the context to the following section “Dynamic Feedback Inhibitory Control”. Placing Figure 1 in the Results would require introducing the regimes of the model without the support of the figure and leave it in the results without a necessary context. Second, we believe that such placement of the figure and accompanying section serves the overall flow of the paper.
We sincerely hope that the reviewer will find our reasoning acceptable.

R3.C2.4
It would be nice to include a scheme or drawing of the model. Perhaps the authors can move the Fig S1 in Supp Material to the Main Text.

Authors’ Response:

Following the reviewer’s suggestion, we placed the schematic of the model in the main text as Fig 1 at line 166.

R3.C2.5
Line 346 in results: “Before convergence of wFIC_i one can encounter...” This may be moved to the Results section.

Authors’ Response:

We have moved “Before convergence of $wFIC_i$ one can encounter damped oscillations towards the dFIC equilibrium. Hence, we choose initial conditions based on the bifurcation structure in order to reduce these transients and obtain faster convergence of $wFIC_i$.” to the last paragraph of “dFIC in a Brain Network Model” (line 525).

R3.C2.6
Line 391: “We have identified...” - move to Results. Figure 2 should be in Results, not Methods.

Authors’ Response:
Following the reviewer’s advice we have reorganized the sections of the Methods and Results dedicated to Poincare Maps. We have extended the results by in-depth description of the reasoning behind the interpretation of the maps in context of dynamical regimes of JR. The mentioned fragment starting with *‘We have identified...’*, is now in the Results at the line 667, where it complements the presented findings. The original **Fig 2** got placed in the Supporting Information (**S1 Fig**). We believe that this organization of the text provides more clarity.

R3.C2.7
Line 398 Fitting methods: how many model realizations (random seeds) did you use for stochastic simulations?

Authors’ Response:

In our original approach we have ran a single simulation changing the random seed for every parameter combination. Knowing the bifurcation diagram structure, having densely scanned the parameter space (30 G times 12 y_0^{target} values), and given that the simulations are long enough for the dynamics to reach a stationary state, we did not consider the need for additional

simulations. Finally, we have performed the bootstrapping analysis described in the manuscript to demonstrate that the MMF fitting results were not random. For that purpose, we have used subsamples of the signals used in our main analysis. This in our opinion constituted the minimal proof of viability of our approach. Nevertheless, the reviewer makes a good point about this possibly being a concern.

Therefore, we have also performed 15 additional simulations with different random seeds for the entire parameter space. Our original best fits based on the MMF value are within one standard deviation of the best fits of those 15 additional simulations (S7 Fig A-B) for the majority of y_0^{targets} and for both post-FIC and no-FIC simulations. The cases for which the best fitting value fell outside the range of one standard deviation ($y_0^{\text{target}} =$ 0.01 and $y_0^{\text{target}} = 0.12$ can be explained by the very low standard deviation (i.e., low variability) among the 15 additional simulations and a wide range of optimal G values yielding similar results. **Fig RV4.** illustrates these results. We decided to add the figure to the Appendix as **Fig A3.**

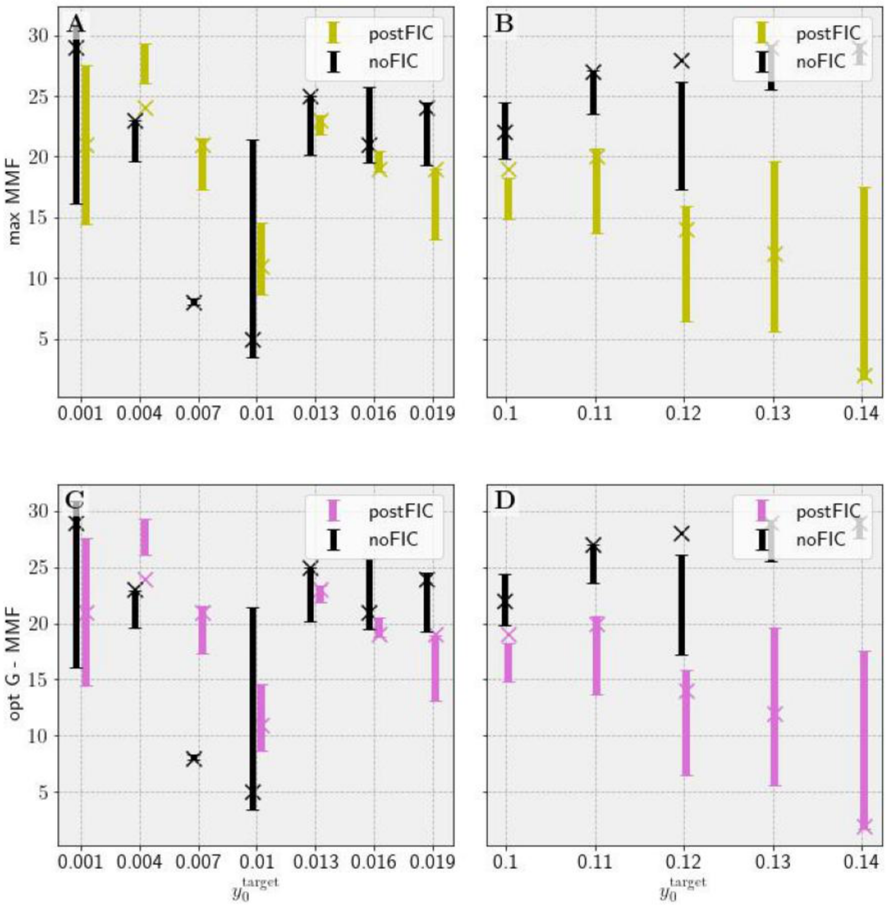

Fig RV4. The results from multiple realization of the MMF fitting per y_0^{target} (A-B) with the corresponding G values (C-D). The optimal values are market with an X and error bars correspond to the standard deviations per y_0^{target} across all simulations.

Additionally, we computed the distance of the best fits of our original simulations to the mean of the 15 additional simulations. The maximum difference between the best fitting values and the mean values of the 15 control simulations is only <10% of the respective mean values. It is worth noting that the standard deviation of the best fitting MMF values in the control simulations is small compared to the mean value i.e., these results are very similar (**Fig RV4**.) This fact provides an additional argument for the robustness of the reported results.

y_0^{target}	post-FIC	no-FIC
0.001	-0.0483801	0.0575306
0.004	-0.0757016	-0.0109205
0.007	-0.0187202	0.0207826
0.01	0.0280787	0.00192505
0.013	-0.0144591	-0.0202474
0.016	0.0185855	-0.00272057
0.0189	0.00056999	-0.00281066
0.1	-0.0173167	-0.00335144
0.11	-0.0254071	-0.026357
0.12	0.0947471	-0.0236518
0.13	-0.0450098	0.0381935
0.14	0.000817855	0.0317354

Table RV1. Relative distance of the best fitting MMF scores to the mean of control simulations per y0_target for both post-FIC and no-FIC simulations. The relative distance is a difference of the best fitting simulation MMF value to the mean of the MMF values from control simulations divided by that mean.

R3.C2.8

Line 413 in Methods: "Into a single multi-modal fitness ..." add the acronym (MMF).

Authors' Response:

The suggested acronym (MMF) has been added to the line 373.

R3.C2.9

Line 429 in Methods: "To test if differential MMF fitting...". That entire part may be moved to Results.

Authors' Response:

We followed this suggestion, and the mentioned paragraph was added to the results section after the fitting results in the line 657.

R3.C2.10

Line 538 in Results: "In summary, the correct application...". That phrase is redundant and can be removed.

Authors' Response:

The suggested redundant paragraph has been deleted.

R3.C2.11

Line 814 in Discussion: "In contrast, when the model was tuned to...". Limit to discuss the results, not repeat them in the Discussion.

Authors' Response:

We agree and replaced repeated results at the line 781 with: *"Predictably, tuning the system to high-activity $y_0^{\text{target}} > 0.11$ values, yielded worse fits than the respective no-FIC simulations, due to all regions being constrained to the fast limit cycle, providing additional argument for the role of dynamical variability in BOLD fitting."* Thus, we now present the interpretation instead of repeating the result.

R3.C2.12

Line 823 in Discussion: "Overall, dFIC gives more...". I think this might be removed.

Authors' Response:

We agreed with this suggestion and removed the sentence.

R3.C2.13

Line 834 in Discussion: “The criticality hypothesis says that...”. This was explained in the Introduction, and the Discussion section

Authors’ Response:

We agreed and removed the sentence.
